# Heart Block (Adams-Stokes Syndrome)

**Published:** 1913-09

**Authors:** 


Heart Block (Adams-Stokes Syndrome). By I. W. Held, *New York Med. Jour.*, 1913, xcvii, 763. Held reviews the experimental work done in heart block, beginning with the experiments of Romanes on the umbrella of the jelly fish and of Tigerstedt, Wooldridge and McWilliams on the mammalian heart in 1884, 1883 and 1888, respectively. He points out that heart block is not synonomous with Adams-Stokes syndrome; that the latter is the clinical manifestation of the former; but that the former may exist without the latter. The following pathological conditions have been described at autopsy: gumma of the bundle of His, stretching and obliteration of the auriculo-ventricular bundle, lymphatic deposits in the bundle, round celled sarcoma of the interventricular septum, infarction of the bundle of His, etc. It is safe to conclude that a slow pulse (the author probably means a constant pulse rate in the neighborhood of 30 or 40 per minute) is due to dissociation between the auricle and ventricle and that the syncopal attacks in Adams-Stokes syndrome are due to the cessation of the ventricular beat; not to the auricular pulsation. In addition to cases of Adams-Stokes syndrome dependent upon disease of the bundle of His we have a type in which the bundle of His is intact but in which the myocardium is extensively diseased; the mural type; and the neurogenic type. The latter type of case is influenced by atropine. Digatalis causes heart-block and whenever the polygraph shows an increased a-c interval that drug should not be used. The prognosis is bad. If the condition is due to a gumma, antiluetic treatment may give brilliant results. Sodium or potassium iodide should be tried in any case. Hydrotherapy is of value; the neutral bath being most efficacious. Carbonated brine baths should be used with great caution and never when there is hypertension. Atropine is useful in the neurogenic type. Caffein and sodium benzoate may be tried. Nitroglycerin should never be used.

